# The black box of the relationship between breast cancer patients and accompanying patients: the accompanied patients’ point of view

**DOI:** 10.1186/s12885-024-12585-z

**Published:** 2024-07-10

**Authors:** Marie-Pascale Pomey, Monica Iliescu Nelea, Cécile Vialaron, Louise Normandin, Marie-Andrée Côté, Mado Desforges, Pénélope Pomey-Carpentier, Nesrine Adjtoutah, Israël Fortin, Isabelle Ganache, Catherine Régis, Zeev Rosberger, Danielle Charpentier, Lynda Bélanger, Michel Dorval, Djahanchah P. Ghadiri, Mélanie Lavoie-Tremblay, Antoine Boivin, Jean-François Pelletier, Nicolas Fernandez, Alain M. Danino, Michèle de Guise

**Affiliations:** 1 Research Centre of the University of Montreal Hospital Centre, Montréal, QC Canada; 2 Centre d’excellence sur le partenariat avec les patients et le public, Montréal, QC Canada; 3 Department of Health Policy, Management and Evaluation, School of Public Health, Université de Montréal, Montréal, QC Canada; 4 CHU de Québec, Université Laval Research Centre, Québec, QC Canada; 5 Centre Intégré Universitaire de santé et services sociaux de l’Est-de-l’Île-de Montréal, Hôpital de Maisonneuve-Rosemont, Montréal, QC Canada; 6 Institut national d’excellence en santé et services sociaux (INESSS), Montréal, QC Canada; 7 Faculté de Droit, Université de Montréal, Montréal, QC Canada; 8 Gerald Bronfman Department of Oncology, Lady Davis Institute for Medical Research, Jewish General Hospital & McGill University, Montréal, QC Canada; 9 Centre Hospitalier Universitaire de Montréal (CHUM), Montréal, QC Canada; 10 Faculté de pharmacie, Université Laval, Québec, QC Canada; 11 Centre de recherche du CHU de Québec-Université Laval, Québec, QC Canada; 12 Centre de recherche du CISSS Chaudière Appalaches, Lévis, QC Canada; 13 Department of Management, HEC Montréal, Montréal, QC Canada; 14 Faculty of Nursing, Université de Montréal, Montréal, QC Canada; 15 Institut universitaire en santé mentale de Montréal, Montréal, QC Canada; 16 Department of Family and Emergency Medicine, Faculty of Medicine, Université de Montréal, Montréal, QC Canada; 17 Centre intégré de santé et de services sociaux de la Montérégie-Ouest, St-Hubert, QC Canada; 18 Yale Program for Recovery & Community Health, New Haven, CT USA; 19 Canada Research Chair in Partnership with Patients and Communities, Ottawa, Canada

**Keywords:** Accompanying patients, Accompanied patient, Peer support, Oncology, Patient care experience, Clinical team

## Abstract

**Background:**

The PAROLE-Onco program was introduced in the province of Quebec, Canada in 2019. It integrates accompanying patients (APs), i.e., people who have been affected by cancer, into the clinical team as full members. These APs use their experiential knowledge with people undergoing treatment and with clinical teams. The aim of this paper is to evaluate, within the framework of two university medical centers, the perceptions of breast cancer patients who receive support from APs, particularly in terms of their active involvement in their care trajectory.

**Methods:**

A qualitative study based on semi-structured interviews with accompanied patients was performed. Fourteen individual interviews were conducted between July and September 2021 with women presenting different profiles in terms of age, education, professional status, type of treatment, family situation, and clinical background. The data were analyzed using thematic analysis, focusing on patients’ perceptions of APs’ contributions and suggested improvements for accessing AP support.

**Results:**

Three themes emerged from the semi-structured interviews: communication modalities used to connect patients with their APs, the characteristics of the support provided by APs, and the perceived effects of this support on the patients. Patients expressed a preference for telephone communication, highlighting its convenience and accessibility. The support provided by APs included emotional and informational support, neutrality, and adaptability. This relationship improved patient communication, reduced anxiety, helped regain control, and enhanced overall quality of life. The results emphasized the added value of APs in complementing the support offered by healthcare professionals. Patients noted the critical role of APs in helping them navigate the healthcare system, better understand their treatment processes, and manage their emotions. The ability of APs to provide practical advice and emotional reassurance was particularly valued. Overall, the findings underscored the significant impact of AP support on patients’ experiences and highlighted areas for enhancing this service.

**Conclusion:**

This study highlights, during the care trajectory of people affected by breast cancer, APs’ contribution to patients’ emotional well-being because they improve, in particular, the management of emotions and communication with health professionals.

**Supplementary Information:**

The online version contains supplementary material available at 10.1186/s12885-024-12585-z.

## Abstract (plain language summary)

In 2019, we initiated the PAROLE-Onco program in Quebec, Canada, to support cancer patients by integrating Accompanying Patients (APs) into the medical team. These individuals, who have personally experienced cancer, join as full team members, sharing their insights with both patients and medical staff. Our study delved into the perceptions of breast cancer patients at two university hospitals regarding APs’ involvement in their care trajectory. Through interviews with 14 women of diverse backgrounds and cancer experiences, we found that APs were instrumental in enhancing communication with doctors, facilitating the expression of challenging emotions, and aiding in treatment decisions. Patients valued the inclusion of APs in their care team and expressed gratitude for their support. Nonetheless, some encountered difficulties in reaching out to APs due to a lack of awareness or challenges involved in connecting. Overall, our research underscores the positive impact of involving APs in the care of breast cancer patients, and of enhancing emotional well-being and communication throughout the treatment journey.

**Supplementary Information:**

The online version contains supplementary material available at 10.1186/s12885-024-12585-z.

## Introduction

Since 2019, two institutions in the province of Quebec, Canada, have introduced accompanying patients (APs), i.e., patients who have already experienced an episode of cancer, into the clinical team and the care trajectory of patients treated for breast cancer. This initiative is part of the PAROLE-Onco program (Patient AdvisoR, an Organizational resource as a Lever for an Enhanced Oncology patient experience), an organizational resource that acts as a lever to improve the experience of oncology patients and professionals [1]. The APs are peers integrated into the clinical teams. They use their experience of living with the disease and navigating health services to guide patients who are receiving an initial breast cancer diagnosis and during their care journey. They also use their own experience to make health professionals aware of patients’ experiences, specifically the difficulties they are undergoing [2]. Of course, studies in the oncology field have highlighted the reality that patients’ emotional and informational support and health professionals’ awareness of patients’ needs are in fact insufficient [3, 4].

Studies indicate that patients with cancer frequently experience feelings of isolation and anxiety and report a lack of empathetic understanding from healthcare providers [5]. This shortcoming has inspired the integration of Accompanying Patients into clinical teams to provide dedicated, empathetic support that complements medical care. This bottom-up approach, supported by healthcare policy shifts towards patient partnership care, has led to the formalization of the AP’s role. By integrating APs into clinical teams, we aim to address the emotional and psychosocial needs of patients, thereby enhancing overall care quality and patient experience [6–8].

Based on these arguments, PAROLE-Onco established the hypothesis that APs can play a decisive role in meeting these needs.

The two health institutions where PAROLE-Onco is implemented have selected, trained and coached APs to intervene with women who are on a breast cancer care trajectory [2]. Patients have the opportunity to meet with an AP, from the start of their care trajectory and on multiple occasions at the start of each of their treatments. The number of meetings between a patient and an AP can therefore vary from one patient to another [9].

If the patient agrees to meet with an AP, the patient’s information and profile are shared anonymously with the APs who then decide, among themselves, which one will accompany the patient in question, based on the similarities between their specific history and that of the patient. The name of the patient is then shared with the specific AP and the AP contacts the patient by telephone to schedule an in-person, videoconference or telephone meeting. After each meeting, the AP completes a logbook which is shared with the clinical team and the patient is kept abreast of the information that is shared. Between March 2020 and December 2021, 124 patients from one institution where PAROLE-Onco was launched and 78 patients from the second institution benefited from this support.

Breast cancer presents a major challenge in terms of emotional support and communication between patients and healthcare professionals [10, 11]. Although accompanying patients (APs) have demonstrated in previous studies their ability to improve communication with healthcare professionals and offer valuable emotional support through their personal experience with cancer, there is still a lack of in-depth research on the direct perception of patients regarding the contribution made by APs [2, 9, 11]. Our study aims to fill this gap by evaluating not only patients’ perceptions of the contribution of APs but also by identifying the improvements needed to optimize the accessibility and effectiveness of this support in the breast cancer care pathway.

This study aims to collect the perceptions of breast cancer patients who have benefited from support sessions with one or more APs in these two healthcare institutionsto evaluate their contribution and the way the intervention was implemented.

## Methods

### Study design

A qualitative study including semi-structured interviews with accompanied patients was conducted between July and September 2022 in the two institutions that implemented PAROLE-Onco. These interviews focused on their perception of the contribution of the APs’ support and on the process of accessing an AP.

### Selection of accompanied patients

Inclusion criteria required French-speaking breast cancer patients with a confirmed cancer diagnosis or who have undergone cancer treatment and have received accompaniment during medical consultations or treatments. Exclusion criteria included individuals unable to comprehend the study’s objectives or provide informed consent, those in critical health conditions, and those with severe cognitive impairments affecting interview responses.

The patient selection process was developed using a methodology that takes into account criteria for diversity in terms of experiences [12]. The steps in this analysis include first collecting information from all patients supported during the period under review while ensuring that duplicates are eliminated for each institution. The information collected focused on the age of the patients, the number of meetings they had, as well as their degree of satisfaction with the support. The data was then sorted according to the different possible combinations that enabled patients to be classified into six different profiles in terms of age, education, professional status, type of treatment, family situation, and clinical background. In total, 53 patients were selected, including 28 from institution 1 (I1) and 25 from institution 2 (I2). Data saturation was achieved with the interviews conducted, as no new significant information emerged from the discussions. Two additional interviews were conducted to confirm this saturation (I1-7 and I1-8) All patients were contacted by two research professionals (MIN and CV) by email. Eight patients from I1 (response rate 29%) and six patients from I2 (response rate 24%) agreed to take part in an interview. The characteristics of the patients are grouped in Table 1. In addition, a summary of the support, family context, and clinical situation of the patients, as well as the number of meetings and the AP assigned to each patient, are presented in Table 2.

**Table 1:** General characteristics of accompanied patients*

Characteristics	I1	I2
Number of patients	8	6
25–34 years old	0	0
35–44 years old	3	1
45–54 years old	2	1
55–64 years old	2	3
65–74 years old	1	1
Born in the province of Quebec	7	5
Born outside of Canada	1	1
College education	1	2
University degree	7	4
Works part-time	0	0
Works full-time	6	5
On leave (illness or maternity)	1	1
Volunteer	0	0
Retired	1	0
*Metastatic breast cancer*		
Yes	1	0
No	6	5
Not responded	1	1
*Stage of care trajectory*		
Before start of treatment	4	1
Full surgery	3	1
Partial surgery	0	4
Reconstructive surgery	1	0
Chemotherapy	2	1
Radiotherapy	0	1
Hormone therapy	1	1

* Patients may have had contact with an accompanying patient (AP) at various points of their care journey

**Table 2:** Summary of the support, family context and clinical situation of the accompanied patients (APs)

Institution (I)	Participant No	Recruitment date	Age group (years)	AP meeting	Number of meetings	Family context	Clinical situation
1	I1-1	20-07-2020	35–44	AP 3	3	Single-parent family	Undergoing treatment (hormone therapy, full surgery)
1	I1-2	22-07-2020	65–74	AP 3	7	Couple without children at home	Before treatment begins
1	I1-3	02-09-2020	55–64	AP 1	10	Single-parent family	Before treatment begins Metastatic cancer
1	I1-4	01-10-2020	45–54	AP 5	1	Couple without children at home	Before treatment begins
1	I1-5	04-02-2021	35–44	AP 4	9	Couple without children at home	Undergoing treatment (parenteral chemotherapy)
1	I1-6	19-01-2021	45–54	AP 3	3	Single-parent family	Undergoing treatment (parenteral chemotherapy, full surgery)
1	I1-7	07-05-2021	55–64	AP 2	7	Couple without children at home	Undergoing treatment (complete surgery, reconstructive surgery)
1	I1-8	19-02-2021	35–44	AP 1 et AP 2	6	Unrelated persons only	Before treatment begins
2	I2-1	10-06-2020	55–64	AP 6	1	Couple without children at home	Undergoing treatment, Hormone therapy Radiotherapy Partial surgery
2	I2-2	30-09-2020	65–74	AP 7	1	Couple without children at home	Undergoing treatment (full surgery)
2	I2-3	06-02-2021	35–44	AP 7	1	Couple with children at home	Undergoing treatment (partial surgery)
2	I2-4	01-03-2021	45–54	AP 8	6	Person living alone	Before treatment begins
2	I2-5	19-03-2021	55–64	AP 8	1	Single parent family	Undergoing treatment (partial surgery)
2	I2-6	09-09-2020	55–64	AP 9	4	Couple without children at home	Undergoing treatment (oral chemotherapy, partial surgery)

### Context of accompanying patients

The selected patients were accompanied by five different APs in I1 and by four different APs in I2, knowing that I1 recruited eight APs and I2 recruited 6 APs. The characteristics of the APs who accompanied the patients in the interviews are presented in Table 3.

**Table 3:** Characteristics of APs

AP record	Institution (I)	Age group (years)	Family context	Clinical portrait
AP 1	I1	45–54	Person living alone	Metastatic cancer Hormone therapy Radiotherapy Partial surgery Reconstructive surgery Immunotherapy
AP 2	I1	55–64	Couple without children at home	BRCA genetic mutation DIEP (deep inferior epigastric perforators) reconstructive surgery Oophorectomy
AP 3	I1	55–64	Couple with children at home	Parenteral chemotherapy Hormone therapy Radiotherapy Partial surgery Reconstructive surgery
AP 4	I1	65–74	Couple without children at home	Radiotherapy Full surgery
AP 5	I1	65–74	Person living alone	Chemotherapy through the veins Hormone therapy Radiotherapy Partial surgery
AP 6	I2	55–64	Couple without children at home	Hormone therapy Full surgery Reconstructive surgery
AP 7	I2	45–54	Couple with children at home	Parenteral chemotherapy Hormone therapy Radiotherapy Full surgery Reconstructive surgery
AP 8	I2	65–74	Person living alone	Parenteral chemotherapy Hormone therapy Radiotherapy or brachytherapy Partial surgery
AP 9	I2	65–74	Person living alone	Hormone therapy Radiotherapy or brachytherapy Partial surgery

### Data collection

The interview guide was established based on our previous research into APs’ perceptions of their contribution [2, 9, 11]. It covers the perceptions of the contribution of the APs’ support at the informational, educational, emotional, and navigational levels and of the process involved in accessing an AP (See Appendix 1: interview guide).

The interview guide was pilot-tested with two researcher-accompanying patients (MD and MAC) to refine the questions and ensure clarity. Following this, all interviews were conducted using the refined guide (see Appendix 1). Interviews with accompanied patients took place between July and September 2022 and lasted an average of 40 min. All participants signed a consent form, and the semi-structured interviews were recorded and transcribed. The interviews were recorded with the participants’ consent. The transcripts were then anonymized to protect the participants’ identities. The interviews were conducted either by telephone or videoconference in French by two research professionals (MIN and CV) between July and September 2022. All digital data was stored on the institution’s server where the research was conducted, secured with a password and user code.

### Data analysis

The data analysis process in this study was guided to assess the perception of the contribution of the APs’ support and of the process involved in accessing an AP. The data are analyzed using thematic analysis, involving phases such as familiarization with the data, generation of initial codes, identification of themes, revision of themes, definition and naming of themes, and production of the report, focusing on patients’ perceptions of APs’ contributions and suggested improvements for accessing AP support. For the data analysis, six steps were followed [13]. The first step consisted of transcribing all interviews (PPC and NA) and directly involving the co-researchers in the coding (MPP, MD, MAC, IF) and reading the interviews to familiarize themselves with the data. The second step led the co-researchers (MPP, MAC, MD) and the research professionals (MIN and CV) to independently code three interviews to extract different themes as part of an inductive approach to allow themes to emerge [14]. The third step involved two meetings between the researchers and research professionals to develop the codebook, which includes four main categories (the type of support, the specificity of this support, the perception of the contribution of the support for oneself, and the venues for improving the contact/access process), and the themes intended to characterize these categories in greater detail. Once the codebook was established, the fourth step enabled the coding of all the interviews in the QDA Miner software (version 6.0.2.) to be conducted by professional research/researcher pairs. In the fifth step, the was continuously refined. The final step consisted of analyzing the results and selecting the most relevant verbatims to illustrate the themes. The verbatims were translated into English and re-transcribed back into French to ensure that the transcription was accurate [15, 16].

### Reflexivity and rigour

The researchers and research professionals had no prior relationship with the accompanied patients interviewed. When selecting participants, all potential candidates were approached, and the participants chosen were those who had agreed to participate first. This research follows the Standards for Reporting Qualitative Research (SRQR) [17]. We implemented ongoing reflective practices to recognize and mitigate any potential influences on data collection and analysis. In addition, a level of rigour was maintained through methodological transparency, including full descriptions of the research process, data collection methods and analysis techniques [18]. To ensure our research was credible and reliable, we followed criteria such as credibility, transferability, dependability and confirmability. We ensure credibility by verifying the data with participants and remaining actively engaged with them over an extended period. Transferability is facilitated through clear descriptions of our methodology. Dependability is maintained through transparent procedures, and confirmability is ensured by considering researchers’ biases. Additionally, we employ member-checking techniques, where participants review our interpretations of their responses, ensuring alignment with their experiences. Moreover, we collaborate closely with patient researchers throughout the research process, leveraging their insights and perspectives to enhance the validity and rigour of our study [19].

## Results

**Fig. 1:**
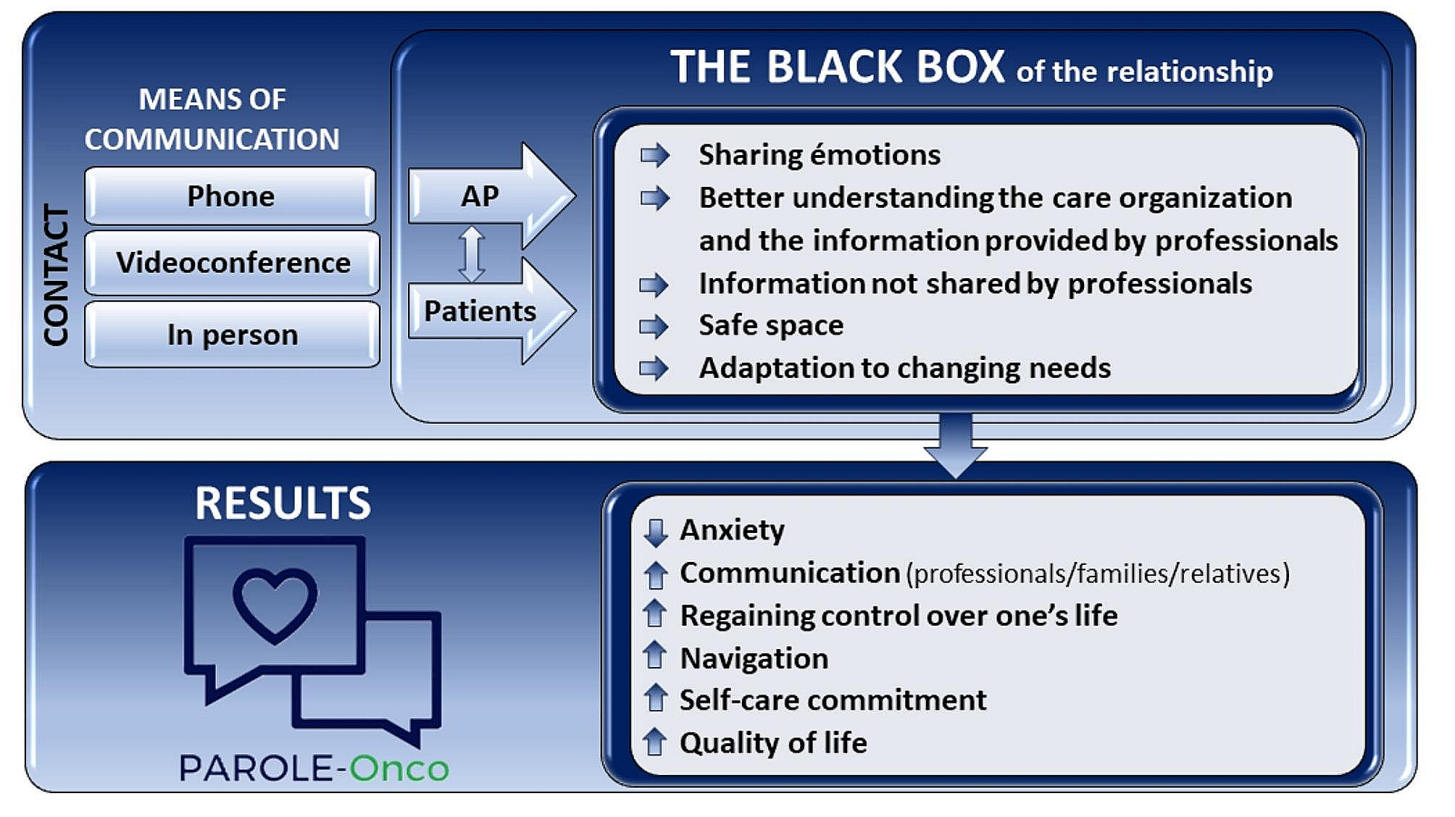
Main themes emerging from interviews with accompanied patients

The results are presented by first focusing on the different communication methods used to put the patients in contact with the APs, and then we looked at the characteristics of support provided by the APs which we referred to as “the black box of the relationship.” We then presented the perceived effects of these meetings on the patients. And, finally, feedback on the accessibility to the APs are discussed, highlighting proposed aspects for improvement. Each verbatim is presented by identifying the institution (I1 or I2) and the interview number. The synthesis of the results are presented in Fig. 1.

### Access for accompanying patients

Both facilities offer several ways for patients and APs to come into contact with each other, including by telephone, by videoconference or in person. Patients highlighted that they appreciated the support over the telephone.

*Basically, as a patient, it is important to ultimately highlight the patient’s testimonial, which is very caring and very personal. Hey, I lived through it, I got through it. The objective is to formulate your request and find a solution so that communication can take place promptly. In a flexible manner, depending on everyone’s schedule, and in particular to organize phone meetings, I will make myself available.* (I2-03)

*It was just over the phone. Well, it was me who said when I wanted to talk, then she* (the AP) *called me. Um, and then asking me questions if I had any questions. But really supported me a lot.* (I1-01)

In addition, some feel that face-to-face meetings are also essential, either in person or by videoconference, especially during a pandemic:

*The phone makes it easier, but I sure would have liked that, at least once, to meet in person.* (I2-01)

*Being sick in times of a pandemic is very lonely. The proposal to organize meetings in person or by videoconference presents itself as a potential response to relieve this negative impact of isolation.* (I1-08)

*And on top of that, it was by phone, which is not always easy because you don’t have the person in front of you.* (I2-02)

*And it was my accompanying patient who called me to give me an appointment, so uh. It was done over the phone every time. We had phone meetings and then it went well.* (I1-06)

*From the initial shock of the news* (diagnostic), *my accompanying patient proved to be an essential support. His* (the AP) *regular telephone contact, weekly or at key times, was particularly soothing and reassuring. His telephone support was very effective, as he instantly dissipated stress, and his kind words were comforting.* (I2-04)

They also appreciate being able to be in contact with the AP several times:

*I enjoyed being able to talk to her* (the AP) *several times.* (I2-06)

*Every time I wanted to talk to her, she* (the AP) *was available.* (I1-07)

*In total, I spoke to her* (the AP) *4 times.* (I1-02)

All patients interviewed appreciate how the support took place: *I appreciate the beautiful receptiveness […].*” (I2-01); even if they are not always aware of what the AP can bring them: *I didn’t know what services she* (the AP) *could offer, what aspects the patient companion can help us with.* (I1-06)

Indeed, patients have identified issues of access to APs and have suggested improvements:

*The way the connection with the accompanying patient is made could be more effective…* (I1-03)

*The way it’s done, we receive a general email to PAROLE-Onco, to which we reply. We never had the accompanying patient’s direct contact.* (I1-05)

*It seems that professionals don’t talk about the program systematically. It would be nice if they integrated it into their practice. I was also thinking that it would be good to have documents available for consultation, so as to know more about the program.* (I2-04)

### The black box of the relationship between the patients and the APs

The analysis of the interviews revealed five distinct characteristics of the relationship between the patients and the APs: emotional sharing; a better understanding of the care organization based on information provided by professionals; additional information shared by the APs that was not provided by professionals; a safe space; and the capacity to adapt to changing needs.

#### Sharing emotions

Emotional support is the support most extensively cited by the interviewed patients. This support translates, first of all, into the ability to share one’s emotions with a person who has gone through the same journey and to feel less alone:

*When I feel like crying, sometimes, when I really feel the emotion, sometimes it… it gets to me, and I say to myself: she* (the AP) *will understand me, she’s been there.* (I1-03)

*Emotions, moods…. on this, I think that it can help […] an accompanying patient will be more attentive to the emotions and the mood of the patient on the other end of the line than… and has more time*. (I1-03)

*It’s such a wonderful support, not only medically but precisely with Parole-Onco, we are not left to ourselves with our emotions*. (I1-07)

This listening validates and normalizes the emotions experienced which often appear to be abnormal by patients:

*I spoke about my fears, the fear of dying, that it paralyzed me, so she* (the AP) *replied […] that I had normal reactions that it was normal for me to be afraid, for me to be agitated, that my sleep was disturbed, she spoke to me about the cycle of emotions […] that I had different phases to go through*. (I1-06)

#### Better understanding of care organization and information provided by professionals

The informational aspect of the meetings between APs and patients proves essential in the process of understanding medical information. This support was reported by 10 patients. In fact, faced with the complexity of the medical vocabulary, APs play a role in making it more accessible: *Of course, we want to talk about our emotions, but here, what we want to know is how to collect information and understand it. And in this context, the accompanying patients help greatly.* (I2-03)

During their discussions, APs and patients consider how care is organized and who does what among the team members to better understand each of their roles and when to consult them. Those discussions help them to navigate their medical journey.

*She* (the AP) *really answered my questions about the organization and the type of care I needed. It really helped me understand how it all works.* (I2-06)

*I felt that with her* (the AP), *I could take the time to be, because she had experienced this too, and to express my fears, to say to myself ‘well, it would be important for you to address this with your nurse, or the surgeon or the anesthesiologist’, she directed me to the right professionals, explaining to me which points are important to discuss with which professional.* (I1-06)

*[…] she* (the AP) *told me that I could call the nurse whenever I needed even if I was more in direct treatment there*. (I1-02)

*She* (the AP) *really answered my questions about the organization […]. Because I was a little confused, should I talk about that with my oncologist, should I talk about that with my surgeon? Should I call the nurse navigator? What I’m experiencing… Should I talk to a psychologist? uh I was a little mixed up so sort of untangling all that, then uh I, I felt like I needed to hear from someone who had gone through that, a testimonial, you know, that the person could tell me well how, how to manage stress, whether it was normal for me to react in such a way, whether it was to legitimize my emotions a little too much there.* (I2-06)

#### Additional information not shared by professionals

APs also provide information that is not exchanged with professionals, such as information that relates to physical appearance: *She* (the AP) *gave me practical advice, first of all, you know… “this kind of lace bra, in this store, you’ll see, you’ll like it.* (I1-04)

APs share practical information learned through their own experiences, such as how to prepare for surgery: *She* (the AP) *helped me a lot in terms of preparing for my surgery […] what clothes to wear, what will it be like in the hospital? What are the steps before the operation?* (I1-06)

They also show that they provide additional help in areas that professionals are unable to cover:

*It was complementary to everything else, complementary to my appointments, uh… my emails or my calls with the oncology surgeons, with the different specialists too, the pivot nurses, the oncology pharmacists, the technologists. So it was, it was complementary. I even think that health professionals have every interest in a program like this continuing because it perhaps compensates for what they don’t have the time to do, or don’t feel like doing.* (I1-05)

#### Safe space

Patients highlighted APs’ neutrality and lack of judgment. In fact, patients are not afraid of being ashamed when talking to an AP and therefore ask all the questions that come to their mind, including very intimate topics.

*I found it useful, I found it to be a good guide to have, a non-judgmental guide, a person who has experienced a similar illness, a similar care trajectory, a treatment plan that includes… At the same time, there was a certain anonymity too.* (I1-05)

Patients emphasize the distinctive value of APs compared to health professionals, commenting that the AP is better equipped to detect difficult times.

*An accompanying patient will be more attentive to the emotions and mood of the patient on the line than… and has more time.* (I1-03)

*It’s such a great support, not only medically but precisely with Parole-Onco, we are not left to our own fates.* (I1-04)

This sharing takes place in a protected and safe space where patients feel confident to share their emotions, and they are not afraid of being judged:

*It was my own little garden; it was something I didn’t need to explain or share or say at length to others. It was just for me, and I liked that.* (I1-04)

*So it’s a bond of trust, which is on another level… A level of empathy also, which is different and higher […] it’s a very personalized support. That’s how I would describe it.* (I1-05)

*We can talk to her about ourselves, about emotions, that’s how we experience it. Then there is listening*. (I2-03)

*It’s important to have this refuge, if I may say so, to be able to… Ventilate (laughs a little), if necessary*. (I1-07)

#### Adapting to changing needs

Patients emphasize the importance of meetings throughout the trajectory with the AP because their needs and questions evolve throughout the process.

*I think that the more I progressed in my journey, the more I realized that it is important to be supported throughout the process because it requires change. My emotional, physical state, and my environment were forced to change. This led me to ask myself new questions.* (I1-08)

To meet these changing needs, patients recommend that meetings with APs be offered from the start of the medical care.

*Meetings should be offered from the start because human interaction from the start is very important so that the patient does not get lost on the medical side. I think it’s good to have access to everything from the start, even if you feel that it’s a lot of things: patient files, websites, videos to watch, but… human interaction like that there? Yeah. Super important.* (I1-04)

*I think it should be offered from the first appointment […] during a first meeting, I can imagine that many patients are very nervous, they may not listen well but, at least at home, with a clear head, they can think about it again and call the person back.* (I2-05)

### The perceived added value of the peer support

Six major contributions emerged from the interviews: to reduce anxiety, to enhance the capacity to communicate, to promote the regaining of control over one’s life, to better use the system’s resources, to be more engaged in one’s care and, finally, to improve one’s quality of life.

#### Anxiety

Participants unanimously expressed that the meetings with the APs had a positive impact on their experience with the treatments by reducing fear and anxiety, thanks to support during difficult times. Participants highlight the beneficial effect of the treatment by mentioning:

*It made my treatment easy*. *I felt much less anxious after talking to him.* (I2-05)

*There is the psychological aspect that it brought me. She really helped me calm down, soothe me, legitimize my state of anxiety, that I had, that it was normal*. (I1-06)

*I had a real lump in my throat, I could hardly breathe. I was super anxious after my diagnosis was announced. The heart-to-heart conversation I had with the PA gradually made my anxieties come out.* (I1-08)

#### Communication

The vast majority of patients highlighted the importance of APs in enhancing their communication with healthcare experts and/or their loved ones.

Patients highlighted APs’ essential role in encouraging them to ask their doctors relevant questions, think critically about the information received, and take notes to ensure their understanding and retention of information exchanged with clinical staff.

*She* (the AP) *opened the door for me to ask questions. It gave me comfort in the possibility of saying: I didn’t understand, can you repeat that? Or just to validate the information that I had… I thought I understood but… It reassured me to know that I could do it, and it wasn’t embarrassing, that it was fine.* (I1-04)

*To guide you in this, to become a partner in your own care, to ask questions, to not hesitate, precisely, to ask all your questions, for fear that “oh maybe the doctor will not like it if I’m asking him that.‘’ No, you really have to ask questions, and his role is to help you with that. Since she* (the AP) *has already gone through all these stages, she already knows a little about what is coming, and how to approach doctors to make informed decisions; that’s what is very important too.* (I2-04)

When it comes to breaking difficult news to family, the accompanying patient expresses the complexity of the situation:

*What I found difficult, for example, was announcing the diagnosis to my 2 daughters. I think it’s a great question because, in fact, that’s what I found the most difficult*. *The AP was the person who helped me realize the importance of talking to them about it and formulating how to talk to them about it.* (I2-01)

*I was very anxious to tell my children that I had been diagnosed with cancer. So I discussed it with my AP, who had had to make the same kind of announcement herself. She was so helpful in finding the right words to tell them.* (I2-04)

#### Regaining control over your own life

These meetings also inspire patients to regain control over their care journey and to actively participate in their own healing process:

*There is a team that takes care of us when we are sick, and then it’s as if we are a little passive concerning all that, but she* (the AP) *was helping me to regain control by giving me technical and psychological information; well, I regained control a little, precisely, and suddenly, I was acting more, you know, and then was more engaged in my care, basically.* (I2-06)

*I felt like I’d lost all control over my life. Through discussion, I realized that I could be more active and less submissive, that I could regain some control over my life.* (I1-02)

But also, this relationship allows us to transmit the energy necessary to fight: *I would almost like to tell you that these meetings saved me. They made me realize that it was worth fighting for.* (I1-03)

#### Navigation

APs allowed patients to better navigate the health system, with the right health professionals by helping them navigate the system more effectively.

*In a pandemic context, we could not be accompanied by a loved one in our appointments, in treatments, so having the accompanying patient is… it becomes a support that is really significant […].* (I1-05)

*She* (the AP) *really answered my questions about the organization […]. The type of support too.* (I1-06)

*I understood the organization of my care better after my discussion with my AP. I didn’t quite understand the sequence, and I didn’t quite understand everyone’s role either. This enabled me to call on my oncology nurse who I hadn’t thought of before.* (I2-05)

#### Commitment to one’s care

The ability to be proactive in one’s care was also widely highlighted by providing additional motivation to stay informed and involved in one’s care journey.

*It’s an encouragement to be alert and involved in your care. This is one more reason to keep yourself informed and stimulate your interest in your care because you share your journey and your impressions.* (I2-05)

*After my conversation with my AP, I dared to ask for a better understanding of my treatments, the side effects and the alternatives I could have. Then I took the time to reflect before making my decision.* (I1-02)

#### Quality of life

The interviews significantly highlighted the positive impact of meetings with APs on their quality of life.

*My meetings with my AP allow me to improve my quality of life; having a patient companion cannot hurt in any case […] it complements the care we receive; it is a complement to everything else. This helps us to be able to experience this ordeal more gently.* (I1-05)

*I only spoke with my accompanying patient once, but that was enough for me. I was very touched by her ability to listen and by her involvement. My quality of life improved a lot afterwards.* (I2-03)

## Discussion

These are the first results from the PAROLE-Onco research project to examine how breast cancer patients perceive the contribution of APs through semi-structured interviews conducted with 14 patients who had interactions with APs. They provide new insights into the black box of the relationship between patients and APs. They provide a better understanding of patients’ interactions with APs, the nature of these interactions, and the perceived impact of these interactions on patients.

### Organizing peer support

Our results show that contact between patients and APs can take many forms within the two healthcare facilities studied, whether by telephone, videoconference, or face-to-face. The ability to offer these different methods is appreciated, as it enables patients to choose the one that suits them best. It also enables the organizations to adapt to changing circumstances, such as a pandemic situation where on-site presence is no longer permitted [20].

However, patients raised the point that the program is not very well known, and that there is a lack of publicity to give it visibility. This finding had already been raised by patients in a previous study [9]. This leads to proposing that communication campaigns be carried out in the institutions. These campaigns could take the form of advertisements on screens in waiting rooms, flyers, or posters. This finding also suggests that the program should be systematically offered to all patients, on a regular basis, throughout their care. It also highlights the importance of a personalized approach, promoting direct communication between the patients and the APs to optimize patients’ ability to access the program and enhance their experience.

### Clarifying the nature of peer support

Previous quantitative studies carried out as part of the PAROLE-Onco program [9] highlight points of convergence with the qualitative results presented here. Indeed, quantitative results showed that when patients had more than one contact with an AP, this improved their ability to manage their emotions, enabled them to be more involved in the decision-making processes, and allowed them to share their diagnosis with family and friends. Contact with APs also helped them to manage their psychological distress at different stages of the care pathway [16–18]. More precisely, the APs, with their personal experience, possess more tools to identify implicit and explicit patient needs. Thanks to a more personal contact and the time invested with the patients, the AP guides the patient toward the appropriate resources, within the institution. Patients may not always be capable of fully assimilating all the information provided to them. Therefore, having the opportunity to discuss with someone who knows the care process allows them to review the information and ensure comprehension. Finally, regarding communication within the family, the AP provides support to patients on how to communicate the diagnosis to loved ones, thereby facilitating these difficult announcement events.

In addition, this qualitative data collection enabled us to specify in greater detail, from the point of view of the APs, the nature of these interactions, which had not emerged in the quantitative study. Primarily, these findings emphasize the excellence of the environment formed between the patients and the APs, and emphasize the deep emotional connection that is formed. This connection, enabled by the sharing of a common experience between two people, makes it possible to mobilize emotions that are difficult to share with people who have not had the same journey. By creating safe spaces of mutual respect, the exchanges allow the patients to let go and share areas of themselves that are otherwise difficult for various reasons (shame, non-recognition, fear of hurting, etc.).

Accessing the practical expertise of APs proves to be a valuable resource, enabling patients to empower themselves during their treatment and legitimize their emotions. The patient’s mental health improves thanks to the emotional support of the AP, who validates the patient’s emotions and experiences.

This safe space allows for a high level of intimacy, even though they don’t know each other and don’t establish a friendly relationship. This support seems to be all the more appreciated because it is offered on demand and is provided in a flexible way over a certain period.

Moreover, this study allows to delve deeper into the black box that is the relationship between accompanied patients and APs, as accompanied patients develop the benefits they derive in a more specific manner (communication with the healthcare team and loved ones; regaining control over their life; enhancing their quality of life), which APs can envisage but not always effectively evaluate [2, 9, 11, 21].

### Perceived effects of peer support

The results helped shed light on some of the perceived effects of encounters with APs by illustrating how these interactions have the power to transform patients’ perspectives and improve their emotional well-being.

These observations are consistent with the literature which indicates that the psychological and emotional support offered by APs can significantly contribute to a reduction in anxiety and depression in cancer patients. Of course, cancer can cause high levels of anxiety, and our results highlight the importance of this type of support by reducing it. Better control of anxiety allows patients to have a better quality of life. These findings are consistent with previous research that emphasizes the value of emotional support for patients dealing with an illness [2, 14, 21, 22].

These results reinforce the importance of support in the context of breast cancer and argue in favor of a partnership approach between patients and the clinical team to develop reciprocal empathy [23].

By expressing their fears, the patients leave space to better engage in their care and thus regain a certain power or control over their lives. As a result, patients become active players in their care process and feel more empowered to take their place alongside professionals to find the best solutions to achieve their life projects. Numerous studies highlight the difficulties cancer patients envisioned when daring to take their place in their care [24]. However, the healing process also involves a feeling of regaining power over one’s situation and having the possibility of acting on one’s health according to the person’s values [25, 26].

The results also show that the inclusion of APs in the clinical teams leads to new communication platforms/opportunities between patients, health professionals, and APs. APs can therefore be seen as conduits between two worlds, namely, the emotional world and the scientific world [27–30]. By achieving a deeper knowledge of the teams in which the patient is treated, the AP promotes the development of a bond of trust between the patients and their clinical team. The clinical team, which knows the APs, can also easily trust them and is willing to hear and integrate the observations of the AP to improve the care process.

### Limitations of the study

The contexts and organization of the two institutions where this study took place present significant differences, thus limiting the generalization of our results. Although we presented the perspective of the accompanied patients on the APs’ roles and the perceived effects of the supports on themselves and the clinical team, it is equally important to evaluate the multicultural aspects and their impact on these supports. These aspects are addressed in another manuscript currently being prepared.

Another point to highlight is the limited number of accompanied patients who accepted our invitation to participate in the interviews. This may have implications for the representativeness of the results. Interviews with accompanied patients were conducted only at the end of the study, and the small sample of patients may influence the generalizability of the findings.

However, it is worth noting that despite this limitation, data saturation was achieved within these two institutions, ensuring comprehensive coverage of the experiences and perspectives of the accompanied patients in these settings.

The credibility and trustworthiness of our results also depend on several factors. Firstly, the qualitative data collection method may be subject to selection and interpretation biases [31]. To enhance the trustworthiness of our conclusions, we employed member checks, as suggested by Guba and Lincoln (1994) [32]. Additionally, it is important to note that a quantitative study on the evaluation of support has already been conducted for one of the establishments whose qualitative results we present here [9].

Moreover, it would also be interesting to explore how APs’ roles could evolve if they were remunerated rather than working as volunteers, as is currently the case. Additionally, it is essential to include the perspectives of clinical teams in future studies.

## Conclusion

This study explores, from the point of view of patients affected by breast cancer, APs’ contribution during their care journey in health institutions. It highlights the different methods of carrying out this support and it lifts the veil on the black box, a space created between the APs and the accompanied patients. It allows us to qualitatively specify the type of support that is offered by APs and highlight certain perceived effects. The patients highlighted the following as the main contributions: reduced anxiety, improved communication with health professionals, regained control over their lives and their care, an ability to engage in their care in front of their health professionals, and ultimately improved their quality of life.

This study holds important implications for both research and clinical practice. In terms of research, our findings underscore the significance of exploring diverse communication methods to enhance support for breast cancer patients and the need to publicize the program, thereby enhancing its visibility. Additionally, our study highlights APs’ pivotal role in facilitating communication between patients and healthcare providers, suggesting opportunities for future research to delve into the impact of companion involvement on patient outcomes and healthcare delivery. From a clinical perspective, our findings emphasize the importance of creating supportive environments where patients feel comfortable expressing their needs and concerns. This underscores the value of implementing tailored support interventions that address the specific needs of breast cancer patients, ultimately contributing to improved patient experiences and outcomes in the clinical setting.

Based on our findings, it is recommended that the important interaction between APs and clinical professionals in cancer treatment be highlighted. APs provide valuable emotional support, assist in clarifying medical information, and facilitate effective communication between patients and professionals. Moving forward, it is essential to delve deeper into the impact of the PAROLE-Onco program on this dynamic and explore strategies to seamlessly integrate APs into clinical teams. Additionally, future research should focus on assessing how this integration enhances the quality of care provided by oncology centers. Longitudinal studies can further elucidate the sustained benefits of AP support on patient empowerment and psychosocial well-being, thereby contributing to the ongoing improvement of patient partnership in oncology settings.

### Electronic supplementary material

Below is the link to the electronic supplementary material.

**Supplementary Material 1**: Appendix 1. interview grid

## Data Availability

No datasets were generated or analysed during the current study.

## Abbreviations

AP: Accompanying patient/peer
CHU: Centre hospitalier universitaire
CHUM: Centre hospitalier de l’Université de Montréal
CISSS: Centre intégré de santé et de services sociaux
CIUSSS: Centre intégré universitaire de santé et de services sociaux
CR‑CHUM: Centre de recherche du Centre Hospitalier de l’Université de Montréal
INESSS: Institut national d’excellence en santé et services sociaux
PAROLE‑Onco: Patient Advisor, an Organizational resource as a Lever for an Enhanced Oncology patient experience
Qc: Quebec
I: Institution
QDA: Qualitative Data Analysis
