# Supplementary material for: The black box of the relationship between breast cancer patients and accompanying patients: the accompanied patients’ point of view
Source: BMC Cancer. 2024 Jul 10;24:822. doi: 10.1186/s12885-024-12585-z (PMC11234724; doi:10.1186/s12885-024-12585-z)
Supplement: Supplementary file 1 — Supplementary Material 1: Appendix 1. interview grid [file 12885_2024_12585_MOESM1_ESM.docx]

Individual interview guide for patients who have received support from APs

1. In what context did you hear about the possibility of talking to an AP? What were your expectations in terms of this meeting? Specifically, in the context of the pandemic?
2. How did the meeting(s) go? Can you tell me about it/them?
3. What did you gain from this meeting?

*These additional questions will help you answer question 3:*

What change(s) did this have on:

- Your quality of life?
- Your emotions/mood?
- Your relationships with healthcare professionals?
- In the context of the pandemic?
- Your commitment to your care?
- Feeling more engaged/empowered? Ability to make a more informed decision?
- How easy it is to say something when you don't understand?
- How easy it is to understand the information you receive?
- Finding it easy to search for information, and identify reliable information?
- Feeling informed?

3.a. Sub-question: In what ways do APs enable you to better understand the information and language used by healthcare professionals (i.e. to make the information more accessible to the general public)? By using more appropriate language? Do you have an example?

1. How did that meet your needs and expectations?
2. What did you like and dislike about the meeting?
3. How would you describe your relationship with the AP (and how would you describe it in comparison with other caregivers)?
4. What suggestions do you have for improving the APs' service offering?
5. Looking back, would you recommend having an AP to anyone else? Do you think this resource should be offered routinely to all patients? For what reasons, and if so, when should such resource be offered?
6. Should this also be offered after acute treatment, when the patient returns to everyday life?
7. Is there anything else you would like to add or ask?

***Thank you very much for participating in this interview.***
